# RELATION BETWEEN ERECTILE DYSFUNCTION AND AMNESTIC MILD COGNITIVE IMPAIRMENT ACROSS TWO TIME POINTS

**DOI:** 10.1093/geroni/igz038.3476

**Published:** 2019-11-08

**Authors:** Richard Vandiver, Michael J Lyons, Kristy Cuthbert

**Affiliations:** Boston University, Boston, Massachusetts, United States

## Abstract

Previous research by the Vietnam Era Twin Study of Aging (VETSA) demonstrated an association between erectile dysfunction (ED) and cognitive functioning. That finding supports a hypothesis that cardiovascular dysfunction may underlie both ED and problems in cognitive functioning. The purpose of the current research was to extend these findings by investigating a putative association between ED and amnestic and non-amnestic mild cognitive impairment (MCI). MCI is of particular interest because of its relationship with Alzheimer’s disease and other dementing illnesses. VETSA is a longitudinal study of twins who served in the US military during the Vietnam conflict (N= 960) consisting of data collected at age 20 (enlistment), age 55 (VETSA 1), and 61 (VETSA 2). The results of the current analyses show that ED is related to both amnestic MCI (p=.032) and non-amnestic MCI (p=.009) at VETSA 1. At VETSA 2, however, the relationship between ED and non-amnestic MCI was no longer significant (p=.751) while the relationship between ED and amnestic MCI was stronger (p=.001). These results are consistent with ED and MCI sharing, to some extent, a common etiology. Vascular dysfunction, which is associated with both ED and MCI, is a plausible mechanism responsible for the observed relationship. These results also highlight the potential role that may be played by ED as an early indicator of cognitive impairment and, perhaps, pre-symptomatic AD.

